# Basic Spatiotemporal Gait Variables of Young and Older Healthy Volunteers Walking Along a Novel Figure-of-8 Path

**DOI:** 10.3389/fneur.2021.698160

**Published:** 2021-06-08

**Authors:** Arturo Zancan, Stefania Sozzi, Marco Schieppati

**Affiliations:** ^1^Independent Researcher, Pavia, Italy; ^2^Centro Studi Attività Motorie, Neurorehabilitation and Spinal Unit, Istituti Clinici Scientifici Maugeri SB, Pavia, Italy; ^3^Istituti Clinici Scientifici Maugeri SB, Pavia, Italy

**Keywords:** spatiotemporal gait variables, walk ratio, linear trajectory, Figure-of-8 path, ageing

## Abstract

**Background:** Locomotion along curved trajectories requires fine coordination among body segments. Elderly people may adopt a cautious attitude when steering. A simple, expeditious, patient-friendly walking protocol can be a tool to help clinicians. We evaluated the feasibility of a procedure based upon a newly designed Figure-of-eight (nFo8) path and an easy measurement operation.

**Methods:** Sixty healthy volunteers, aged from 20 to 86 years, walked three times at self-selected speed along a 20 m linear (LIN) and the 20 m nFo8 path. Number of steps, mean speed and walk ratio (step length/cadence) were collected. Data were analysed for the entire cohort and for the groups aged 20–45, 46–65, and >65 years.

**Results:** There was no difference in mean LIN walking speed between the two younger groups but the oldest was slower. During nFo8, all groups were slower (about 16%) than during LIN. Cadence was not different across groups but lower during nFo8 in each group. Step length was about 8% shorter in the two younger groups and 14% shorter in the oldest during nFo8 compared to LIN. Walk ratio was the smallest in the oldest group for both LIN and nFo8.

**Conclusions:** A complex nFo8 walking path, with fast and easy measurement of a simple set of variables, detects significant differences with moderate and large effects in gait variables in people >65 years. This challenging trajectory is more revealing than LIN. Further studies are needed to develop a quick clinical tool for assessment of gait conditions or outcome of rehabilitative treatments.

## Introduction

Every day we link up linear walking with turns and circular paths when we move at home or in open spaces, so that a fair proportion of the walking time is spent along curved trajectories (1). Hence, the central nervous system is forced to exert continuous control onto the production of locomotor movement, since our inherently unstable bipedal gait (2) becomes even more critical during turns and curved walking (3).

Successful locomotion along curved trajectories requires fine control to adapt gait to the environmental constraint (4). Curved paths imply extra- and intra-rotation of leg and foot in order to accurately place the foot on the ground (5–9) and accurate coordination of lower limb segments, between limbs, and between limbs and trunk in both the sagittal and frontal planes (10, 11). Definite features have been found in muscle synergies during curved walking (12, 13).

Depending on the radius of curvature of the path and the walking velocity, the trunk and the entire body incline toward the centre of the trajectory: a centripetal force is thereby produced in order to avoid going off on a tangent (14). In order to do so, the peak pressure point of the foot sole during heel strike and toe off is displaced in the frontal plane with respect to its position in linear walking. This creates a mediolateral torque generated by gravity that allows smooth progression along the circular trajectory [see (15)].

Age-related changes in the timing and sequencing of body segment reorientation have been described. Aged or diseased people show turning difficulties, opt for a more cautious attitude when steering and have a higher risk of falling while walking (16–20). U-turning and returning to the initial position (21) or sharp turnings represent critical locomotor phases (22), since they imply rapid lateral translation of the body in addition to reorientation and alignment with the next travel direction (9). Patients with Parkinson's disease walk slowly during curved walking (23), and turning while walking frequently provokes freezing (24). Patients with dementia also show slowing in both progression and performance in cognitive tasks while walking along curved paths (25).

Assessing the ability to walk along non-linear trajectories should be a standard procedure in many clinical circumstances where problems in locomotion become an issue, or to predict impending or future difficulties in old people (26). However, description, analysis and quantification of gait (17, 27, 28) may not be within reach of every rehabilitation facility or gym training location, more so for complex trajectories (29). Besides, the preparation of the participants may not be rapid, and lengthy procedures are not easily accepted by older healthy volunteers or patients. Figure-of-eight trajectories, supposed to simulate activities of daily living, have been exploited for routine evaluation of basic capacities for moving around in an “ecological” way. In those cases, the person has to freely turn around two cones placed on the floor at a certain distance (normally short, about 1.5 m), but several variants of this shape are described (30–35) with no path constraints and no sharp turns.

**Figure 1:**
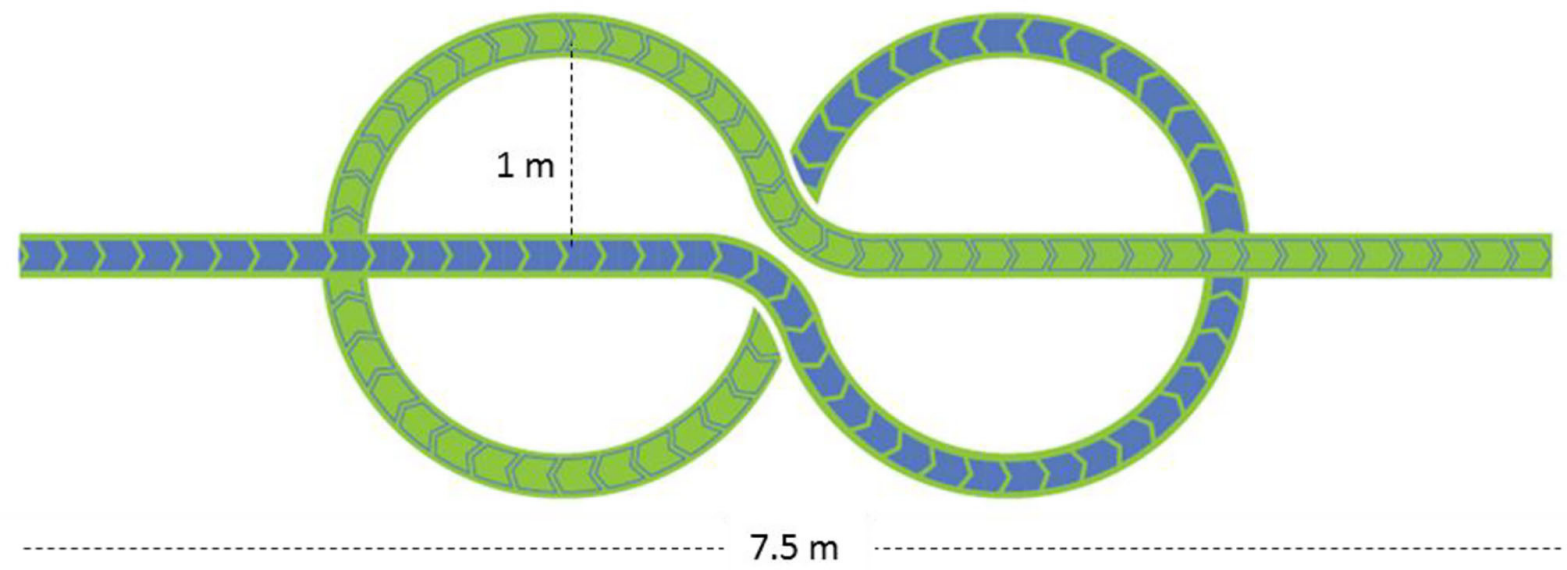
The novel Figure-of-eight (nFo8) path. The path, printed on a large plastic carpet, is composed of a linear walking section (3.7 m) followed by two curvilinear sections (each with a radius of 1 m) and by a second linear section.

Here, we have designed a novel Figure-of-eight (nFo8) path composed of linear and curved trajectories, with two sharp (90 deg) turns and two circles, having a total length of 20 m (see Figure 1). We have assessed the feasibility of this protocol and its ability to rapidly give information about the gait steering competence of typical old able-bodied participants by means of an extremely simple and expeditious procedure. We hypothesised that a particular standardised path containing delineated walking segments would be sensitive to the necessary adjustments of leg and foot orientation, trunk inclination and postural changes compared to linear walking, in particular as age increases. To this end, simple spatiotemporal variables while walking along the nFo8 trajectory have been measured in healthy volunteers of different age, including a group of little studied middle-aged adults (26), and compared with those obtained while walking along a linear path.

## Materials and Methods

### Subjects and Design

Participants aged 20–86 years were consecutively admitted to the study. Recruitment was closed when 20 participants per age group were collected [20–45 years, mean 32.6 ± 6.5 years ± SD, 15 females (F) and 5 males (M); 46–65 years, mean 54.1 ± 5.4, 8 F and 12 M; 65–86 years, 74.8 ± 4.1, 9 F and 11 M]. All volunteers were evaluated at the Istituti Clinici Scientifici Maugeri of Pavia. They were invited to participate if they were able to ambulate independently, had no history of falls and did not take drugs acting on the central or autonomic nervous system. Subjects were excluded if they had vision disorders, clinically evident cognitive problems or impairment in understanding simple instructions, muscle-skeletal soreness or other impairments which could affect balance or gait. The Ethics Committee of the ICS Maugeri approved the study (n. 806 CEC) and informed consent was obtained from all participants.

The two trajectories consisted of a 20 m long straight path and of the 20 m long novel Figure-of-eight (nFo8) path (Figure 1). In the linear (LIN) test, participants walked down a hallway of the hospital ward (overall length more than 40 m, 3 m wide, containing no furniture). The nFo8 path was printed on a thin (<0.5 mm), sturdy roll-up plastic carpet (Arti Grafiche Fimognari, Milano, Italy) laid on the laboratory floor (in an ample laboratory space allowing for several steps at the end of the recorded epoch). The nFo8 path was composed of a linear-walking section (3.7 m) followed by two curvilinear sections (two connected circles, each with a radius of 1 m, allowing for prolonged counter-clockwise and clockwise turning) and a second linear section (3.7 m). The nFo8 path had a width of 20 cm, and the participants placed the feet within the width or on the borders of the outlined strip with no special effort, thereby enabling to reliably estimate the walking speed by dividing the overall path length by the time to cover the distance.

Both walking conditions were tested in a single session. A series of four walking trials for each condition (LIN, nFo8) were performed in sequence by the participants, with at least 2 min interval between each test. Subjects, wearing their usual shoes, performed the two series, starting with the LIN or with the nFo8 condition. For each series, the first of the four trials (a “familiarisation” test) was discarded from the analysis. The participant was placed at the beginning of each 20 m path. At the operator's signal, the participant started walking with eyes open from the standing position at his/her own spontaneous speed and data recording started. Subjects were invited to continue walking a few steps after the end of the path. Time in seconds was recorded, always by the same operator, by means of a stopwatch that started with the onset of the first step (at the “go” signal) and stopped at crossing the target line. Care was taken and instruction given to avoid any “reaction-time” abrupt initiation and no encouragement was given to take the least time possible to complete the task.

### Outcome Measures and Data Analysis

The mean speed (m/s) was calculated by dividing 20 m by the time to cover the walking distance. The number of steps was also counted by the operator, and the mean step length (cm) computed by dividing the walking distance by the number of steps. The walk ratio (step length/cadence) was also calculated. All data were tabulated into an Excel® sheet for further analysis.

The walking speed of the three successive trials were compared with a 3 (age groups) × 2 (nFo8, LIN) × 3 (successive trials) repeated-measure analysis of variance (rm-ANOVA). The mean values ± SDs of the variables recorded while the participants walked along the linear and the nFo8 path were calculated. The data were compared with the use of a 3 (age groups) × 2 (nFo8, LIN) rm-ANOVA. In order to assess gender effects, a 2 (M, F) × 2 (Fo8, LIN) rm-ANOVA was run. The *post-hoc* test analysis was the Fisher's LSD test, with *p* < 0.05 considered for significance. The Cohen's *d* values highlighted the strength of the differences (with *d* = 0.2, 0.5, 0.8 considered small, medium and large effect sizes, respectively) (36). The minimum effect size detectable in the gait speed variable between LIN and nFo8, given our sample size (*n* = 60), was calculated (37). Regression lines were also determined for cadence, step length, walking velocity and walk ratio, all participants' data collapsed, for both walking paths (nFo8 and LIN) and plotted against the age of participants.

Statistical analysis was performed with the software Statistica® (StatSoft, Tulsa, OK, USA). The regression lines (slope and intercept) were compared by means of the Compare Linear Fit Parameters routine of the software Origin® (OriginLab Corporation, Northampton, MA, USA).

## Results

### Walking Speed

**Figure 2:**
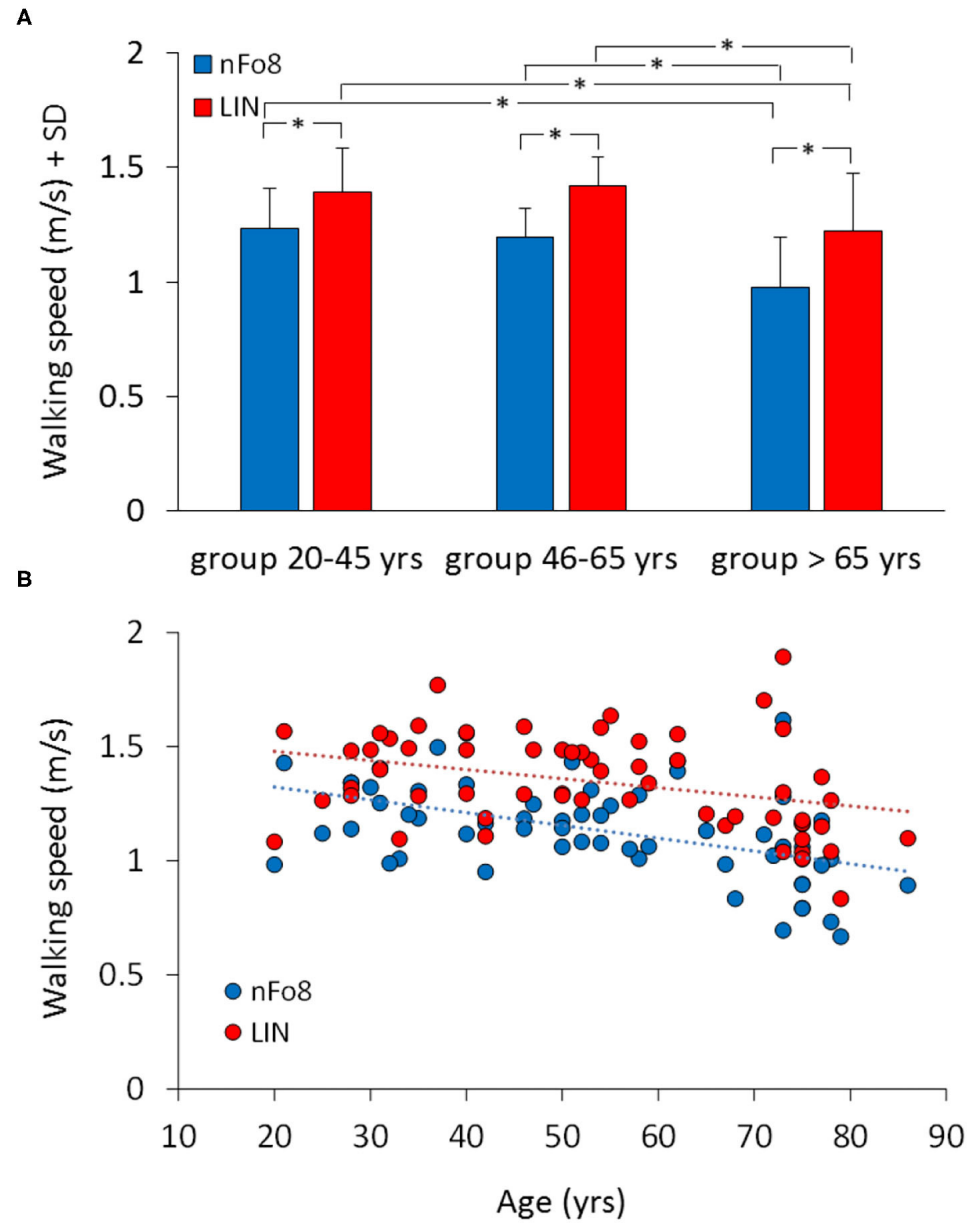
Mean walking speed of the three age groups **(A)** and the relationship between walking speed and age across all participants **(B)**. Blue bars and dots refer to the nFo8, red bars and dots refer to LIN. In all the three age groups, speed was lower during nFo8 than during linear walking. Gait was slower in oldest group than in the young and middle-age group under both walking conditions. Asterisks indicate significant differences.

There was no significant difference among the three successive walking trials following the familiarisation trial, for any age group or trajectory [main effect of repetition, F_(2,114)_ = 2.38, *p* = 0.1]. Figure 2 shows the effect of the two different trajectories (LIN; nFo8) on the mean walking speed across all participants clustered in age groups. Speed was lower during nFo8 than LIN walking (84% of LIN on average) and significantly different between the two trajectories [F_(1,57)_ = 198.15, *p* < 0.0001, *d* = 3.65]. There was a significant difference between age groups [F_(2,57)_ = 9.4, *p* < 0.001, *d* = 1.06]. The age-trajectory interaction was close to significance [F_(2,57)_ = 0.64, *p* = 0.053]. The *post-hoc* test showed that there was no difference in walking speed between the young and middle-age groups during LIN or during nFo8 (*post hoc, p* > 0.6 for both comparisons). However, the older group was significantly slower than the young and middle-age groups for both trajectories (*post hoc, p* < 0.05 and *d* > 0.97 for all comparisons). All participants collapsed, there was no difference in gait speed between males and females [F_(1,58)_ = 1.62, *p* = 0.21] and no interaction with the trajectories [F_(1,58)_ = 0.19, *p* = 0.67]. Given the sample size of 60 participants, the study proved to have a sufficient power (>80%) to detect an effect size in walking speed larger than 0.12 m/s between the nFo8 and LIN trajectories.

The lower panel of Figure 2 shows the relation between walking speed and age across all participants (regression lines, nFo8: *y* = −0.006 *x* + 1.4, *R*^2^ = 0.24, *p* < 0.0001; LIN: *y* = −0.004 *x* + 1.6, *R*^2^ = 0.12, *p* < 0.01). The slopes of both regression lines were not different from each other (*p* = 0.4). Conversely, the intercepts with the ordinate were significantly different (*p* < 0.001) reflecting the differences between the bars in the upper panel.

### Cadence

**Figure 3:**
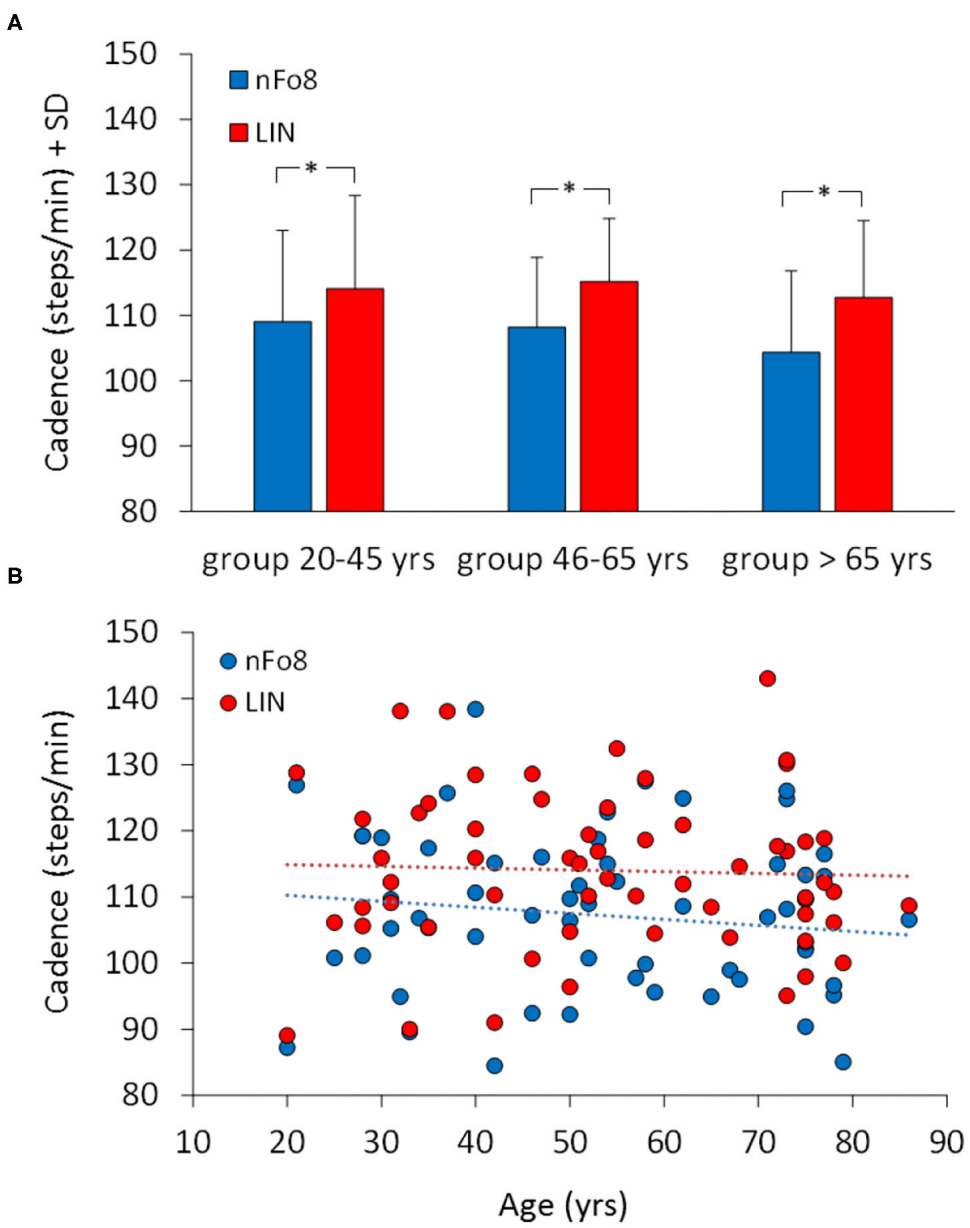
Mean cadence in the three age groups **(A)** and the relationship between cadence and age across all participants **(B)**. Blue bars and dots belong to the nFo8, red bars and dots to the LIN. Cadence was lower during nFo8 than during linear walking in all the three groups of age. Asterisks indicate significant differences.

Figure 3A shows the effect of the two trajectories on the mean cadence. During the nFo8 walking, the mean cadence was about 6% lower with respect to the linear walking. The difference in cadence between trajectories was significant [F_(1,57)_ = 31.2, *p* < 0.001, *d* = 1.43]. There was no significant difference between age groups [F_(2,57)_ = 0.49, *p* = 0.62] and no significant age-trajectory interaction [F_(2,57)_ = 0.64, *p* = 0.53]. The *post-hoc* test showed that there was a significant difference in cadence between LIN and nFo8 in each of the three groups (*post hoc, p* < 0.05, *d* > 0.45, for all comparisons). All participants collapsed, there was no difference in cadence between males and females [F_(1,58)_ = 1.3, *p* = 0.26] and no interaction with the trajectories [F_(1,58)_ = 0.17, *p* = 0.68].

Figure 3B shows that there was no relation between cadence and age across all participants. The regression lines fitted to the data of the two walking conditions (nFo8: *y* = −0.091 *x* + 112.1, *R*^2^ = 0.018, *p* = 0.31; LIN: *y* = −0.026 *x* + 115.4, *R*^2^ = 0.001, *p* = 0.76) were not significantly different from the horizontal. The two regression lines had no different slope (*p* = 0.6). The intercepts with the ordinate were significantly different (*p* < 0.05).

### Step Length

**Figure 4:**
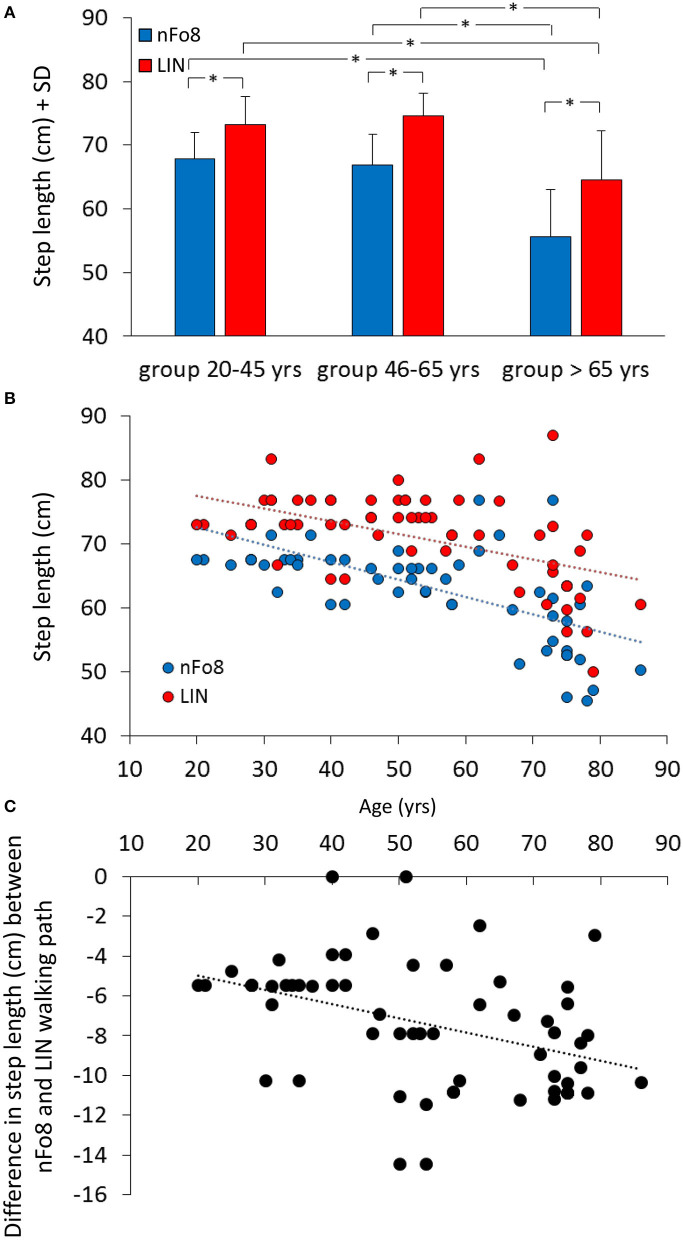
Mean step length **(A)** and the relationship between step length and age **(B,C)**. Blue bars and dots refer to the nFo8, red bars and dots refer to LIN. Step length was shorter during nFo8 than during LIN in the three groups of age. Asterisks indicate significant differences. **(B)** shows the relationship between step length and age: the step length diminished with age for both nFo8 and LIN walking path. **(C)** shows the relationship with age of the individual differences in step length between the nFo8 and LIN walking paths. The difference (shorter step length with nFo8) increased clearly with age.

During the nFo8 walking, step length was about 10% shorter with respect to LIN (Figure 4). The difference between trajectories was significant [F_(1,57)_ = 403.7, *p* < 0.0001, *d* = 5.22].

The mean step length was different across age groups [F_(2,57)_ = 25.6, *p* < 0.0001, *d* = 1.81] and there was a significant age-trajectory interaction [F_(2,57)_ = 7.8, *p* < 0.001, *d* = 0.96]. The step length was shorter in the group > 65 yrs with respect to the other two groups for both trajectories (*p* < 0.001, *d* > 1.38, for all comparisons). In each age group, step length was shorter for the nFo8 than for the LIN trajectory (*post-hoc, p* < 0.0001, *d* > 1.84, for all comparisons). All participants collapsed, there was no difference in step length between males and females [F_(1,58)_ = 0.67, *p* = 0.42] and no interaction with the trajectories [F_(1,58)_ = 2.04, *p* = 0.16]. Figure 4B shows the relation between step length and age across all participants (regression lines, nFo8: *y* = −0.27 *x* + 78.1, *R*^2^ = 0.39, *p* < 0.0001; LIN: *y* = −0.2 *x* + 81.6, *R*^2^ = 0.27, *p* < 0.0001). The slopes of the two regression lines were not different (*p* = 0.25) but the intercepts were significantly different (*p* < 0.001). The bottom panel (c) shows the relationship between the difference in the step length of each participant walking along the two paths (nFo8 minus LIN) and age (*y* = −0.07 *x* – 3.5, *R*^2^ = 0.17, *p* < 0.01).

### Walk Ratio

**Figure 5:**
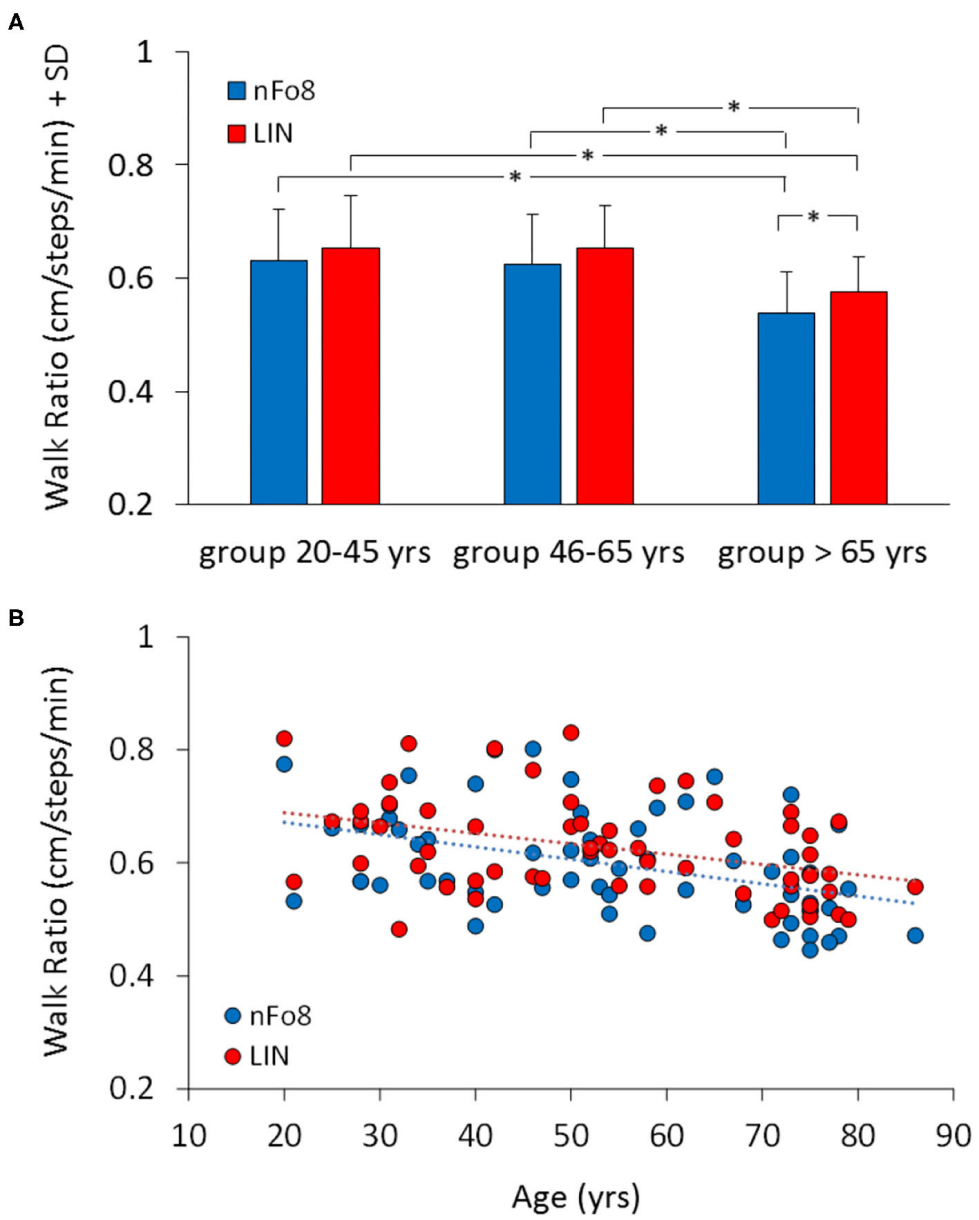
Mean Walk Ratio (WR) **(A)** and the relationship between WR and age across all participants **(B)**. Blue bars and dots refer to nFo8, red bars and dots to LIN walking path. WR was just a little smaller in the group >65 yrs than in the other two age groups. Asterisks indicate significant differences.

When the mean walk ratio (WR, step length/cadence) of the three age groups were computed (Figure 5), the rm-ANOVA found a significant difference in WR between the two trajectories [F_(1,57)_ = 12.2, *p* < 0.001, *d* = 0.87]. In absolute terms, the difference between the two means amounted to about 0.03, i.e., 0.63 (LIN)−0.60 (nFo8).

There was no age-trajectory interaction [F_(2,57)_ = 0.38, *p* = 0.68]. WR was significantly different between age groups [F_(2,57)_ = 7.9, *p* < 0.001, *d* = 0.96]. It was similar for the young and middle-aged participants (*post-hoc, p* > 0.7), but just smaller in the older than in the other groups (*post-hoc, p* < 0.05, for both comparisons for both trajectories). Further, in the older but not in the young and middle-age group, WR was smaller for the nFo8 than LIN path (*post-hoc, p* < 0.05, *d* = 0.75). All participants collapsed, there was no difference in WR between males and females [F_(1,58)_ = 0.03, *p* = 0.85] and no interaction with the trajectories [F_(1,58)_ = 0.001, *p* = 0.98].

There was no age-trajectory interaction [F_(2,57)_ = 0.38, *p* = 0.68]. WR was significantly different between age groups [F_(2,57)_ = 7.9, *p* < 0.001, *d* = 0.96]. It was similar for the young and middle-aged participants (*post-hoc, p* > 0.7), but just smaller in the older than in the other groups (*post-hoc, p* < 0.05, for both comparisons for both trajectories). Further, in the older but not in the young and middle-age group, WR was smaller for the nFo8 than LIN path (*post-hoc, p* < 0.05, *d* = 0.75). All participants collapsed, there was no difference in WR between males and females [F_(1,58)_ = 0.03, *p* = 0.85] and no interaction with the trajectories [F_(1,58)_ = 0.001, *p* = 0.98].

The lower panel B of the Figure shows the relation between WR and age across all participants (regression lines, nFo8: *y* = −0.002 *x* + 0.7, *R*^2^ = 0.17, *p* < 0.001; LIN: *y* = −0.002 *x* + 0.7, *R*^2^ = 0.16, *p* < 0.01). Slope and intercept with the ordinate of the two regression lines were not different (*p* > 0.05, for both comparisons).

## Discussion

This pilot study assessed whether a novel Figure-of-8 (nFo8) walking path, containing straight and curved segments and sharp turns and amounting to 20 m length overall, is able to detect changes in locomotor performance of healthy aged participants walking along complex trajectories. The analysis was based on the simple comparison of basic spatiotemporal variables obtained for the linear (LIN) and nFo8 trajectories and in three groups of participants (young, middle-aged and older). The underpinning stipulation of the study protocol was that the variables should have been obtained easily and promptly by one operator and by means of a stopwatch.

Owing to the patterned nFo8 trajectory, having a width of the walking strip of 20 cm, all participants succeeded without effort in placing the footsteps within the borders of the sketched path. This allowed to calculate the walking speed along the nFo8 as much as along the LIN path. A walking path proposed by Belluscio et al. (28), composed of two large circles, allows the calculation of gait speed as well. Having a measure of walking speed is important, because it is the basic variable in gait assessment under simple and complex conditions (38) and is commonly used for comparison of gait across participants' age and patients' conditions (39).

### Gait Speed, Cadence, Step Length

Walking velocity and step length significantly diminished in the oldest group, as in most studies mentioned above. Overall, gait speed was lower and step length shorter along curved than LIN trajectory as has been shown before (7, 8, 40). The differences between LIN and nFo8 were the largest for the group of participants older than 65 years. The scatterplots of gait speed and step length vs. age clearly showed a cluster of mostly smaller values in the older group for both LIN and nFo8, but the decrease was larger in the nFo8 than LIN condition. A reduction in speed along the circular segments, combined with the placement of the feet at the sharp turns implying control of trunk inclination during the task, may be liable for the overall slowing of progression and changes in the spatiotemporal variables. In the older group, this slowing would indicate a prudent approach to the turns (16), a circumstance requiring subtle control of the inertial forces (41).

Overall, in the LIN path, the mean cadence was about 114 steps/min, the mean gait speed about 1.4 m/s and the mean walk ratio about 0.63 cm/steps/min. These values are comparable to those of the literature for populations in the same age span, taking into account the differences in path length such as 20 m (42); 4 m (43); 5 or 6 or 7 m (44–46); 10 m (47). Older participants tended to have a lower gait speed as already shown for shorter trajectories (48). Of note, in many studies, cadence during LIN remained constant across age groups and diseases [e.g., (40, 44, 49, 50)].

In our study, there was a lower cadence across the three age groups (20–45, 46–65, and 65–86 years) when participants walked along the nFo8 trajectories. This is in keeping with (23) for a group of healthy elderly, even if the trajectory was not exactly the same. Here, the decrease in cadence is likely due to the challenging experimental setup and the cautious gait (26) [one sharp 90 deg turn, 1 m-radius of curvature for the two circles performed in clockwise (CW) and counter clockwise (CCW) direction in sequence, overall amounting to 12.5 m, another sharp turn]. Of note, a radius of 1 m requires a body inclination toward the interior of the trajectory of about 4 deg with respect to the vertical (14). The gait cycle might have been just longer around the two sharp turns and when reversing trunk inclination passing from the CCW to the CW circle.

### Walk Ratio

The WR (step-length/cadence) is a robust index of walking coordination and control (51). It has been suggested long ago that WR can be exploited for evaluating the effects of age and disease on the locomotor pattern (52). WR is normally invariant across walking velocities. During treadmill walking at comfortable speed, WR is independent of age as well (53). In ample cohorts of healthy young or elderly participants walking straight (54, 55), the WR proved to be very close to that obtained here during LIN, where WR was constant in the young and middle-aged participants. The nFo8 slightly reduced the WR in these groups, but the effect was not significant. Conversely, in the older group, WR modestly but significantly diminished during the nFo8 path. The decrease in speed would not be accountable for the diminished WR, because only very low speeds affect WR (56). Of note, the mean decrease in the mean value of step length along the nFo8 path value takes into account the obvious fact that the step length of the leg inside the curved trajectory is necessarily shorter than that of the outer leg (7, 8). Hence, the WR value in the nFo8 condition is affected by the reduction in the *mean* step length along the path.

Oddly, WR is high during dual-task when walking straight on an uneven surface (57). This cannot be compared to our nFo8 condition, where WR diminishes in the older group. This weakens the possibility that walking along the nFo8 requires an enhanced level of attention, as might be supposed due to the geometrical complication of the path and the required changes in muscle activity (58, 59). It is an open question whether the diminished WR in the older group during nFo8 (with a gentle decrease in cadence and a marked shortening in mean step length) can be a sign of initial problems in the independent neural control of each limb. In the study by Nakakubo et al. (60), a small WR is associated with falling in the past year in a cohort of a community-dwelling elderly people, even if these had no slowing of gait speed. In patients with multiple sclerosis, an association was found between the volume of the cerebellum and walk ratio (61).

## Limitations

Differences in height and body weight have not been taken into account, and old-old frail participants were not included in the study. Another limitation consists in the missing comparison of the effects of age on the walking speed between the present nFo8 path and the simpler, free walking Fo8 path(s) set by two traffic cones, which has been a common procedure for gait evaluation [e.g., (30, 62)]. Such comparison could enhance our assessment of the advantages of the nFo8 walking path in the older and the patient, but would not be fully congruous because the trajectory is obviously not constrained in the free, short-length, cone-based Fo8 paths. An important limitation consists in the use of only manual measurement (mean speed and number of steps was counted by one operator). No instrumental outcome measures have been gathered nor a video was taken of the performance in order to check the accuracy of these measurements. Moreover, head and eye movements were not recorded. Gaze redirection may be an essential subcomponent to steering, so that visual and/or oculomotor deficits should be considered when assessing steering behaviour (63), because gaze control can be altered in the elderlies (64). Anyhow, when asked at the end of the walking trials, neither young or older participants mentioned any intentional visual guidance of the walking performance, probably because the path was highly contrasted with respect to the floor and an intermittent quick glance at the curved trajectory at successive time-instants was enough to proficiently progress along the path (65).

## Implications and Perspectives

Walking performance was characterised here by a few parameters. This type of measurement (timed performance and step number) would be very easy to implement in the clinic. Such a simple approach can smartly highlight meaningful interactions between age and trajectory pattern. On average, the time to cover the trajectories was about 14 and 18 s, for LIN and nFo8, respectively. The spatiotemporal variables of the group of healthy participants older than 65 years were definitely outlined by the nFo8 compared to the younger group. The present LIN and nFo8 paths might be more dependable for gait velocity assessment than the often used distance of four or ten metres [e.g., (43, 66)] for durations of only about 4–9 s (48). Subjects reported no unpleasant sensations such as dizziness when walking along the nFo8 path. The distances covered did not produce physical discomfort, and no participants in any age-group declared fatigue or leg tiredness (67). The inclusion of middle-aged persons, which are rarely considered in these studies (68), the comparison between continuous 20 m floor-walking along straight and complex path (including linear and curved segments and two sharp turns) and the simple and expeditious mode of collecting important information appear to have received little attention so far.

Contrary to linear trajectories, the sharp turns included in the nFo8 path require accurate and coordinated activity of the leg intra- and extra-rotator muscles (69–71), which produce pelvis and trunk rotation over the stance leg (5, 72). While manual measurement of leg rotator muscles bears a considerable error (73), a tool such as the nFo8 path would functionally quantify sensorimotor limitations in walking and turning and represent a good screening tool in frail elderlies and patients with locomotor impairment. Further, in older adults, poor executive function would manifest itself more clearly during the nFo8 than other types of Fo8 paths. Similar standpoints become evident in a recent review (74) that supports the superiority of dual tasks associated to turns and other transfer patterns during gait for fall prediction in adults over 65 years of age.

Neither temporal patterning of motor primitives nor muscles synergies seem to be significantly affected by ageing (75), but spinal maps differ significantly between young and older humans depending on the biomechanics of the locomotion. Hence, complex walking paths require a more elaborate activity and descending control of the spinal circuits assisting locomotion, thereby producing slower progression during the nFo8 in older participants (76).

Further research is necessary to assess whether this nFo8 path might help identify gait abnormalities in patients with different ailments affecting turning while walking (77, 78), allow an early differential diagnosis in patients with locomotor impairment of different nature (79, 80), and perform rapid and reliable quantitative assessments with disease progression [see (81, 82)].

## Conclusions

The present preliminary results show that it is possible to quickly assess basic spatiotemporal gait variables in healthy volunteers walking along a complex path including continuous turning. The results also show that the nFo8 path is able to pose a significant challenge to older participants. These preliminary findings warrant further investigation aimed at defining the psychometric properties of this protocol in the context of a cross-sectional study based on a larger number of participants.

## Data Availability Statement

The original contributions presented in the study are included in the article, further inquiries can be directed to the corresponding author/s.

## Ethics Statement

The studies involving human participants were reviewed and approved by the Ethics Committee of the ICS Maugeri (n. 806 CEC) and informed consent was obtained from all subjects. The patients/participants provided their written informed consent to participate in this study.

## Author Contributions

AZ and MS conceived the idea and designed the experiments. SS performed the analysis and the statistics. MS wrote a draft of the manuscript. All authors contributed to the data interpretation and to the final manuscript.

## Conflict of Interest

The authors declare that the research was conducted in the absence of any commercial or financial relationships that could be construed as a potential conflict of interest.

## References

[1] Glaister BC Bernatz GC Klute GK Orendurff MS. Video task analysis of turning during activities of daily living. Gait Post. (2007) 25:289–94. 10.1016/j.gaitpost.2006.04.003 16730441

[2] Ivanenko YP Dominici N Cappellini G Di Paolo A Giannini C Poppele RE. Changes in the spinal segmental motor output for stepping during development from infant to adult. J Neurosci. (2013) 33:3025–36. 10.1523/JNEUROSCI.2722-12.2013 23407959 PMC6619203

[3] Godi M Giardini M Schieppati M. Walking along curved trajectories. Changes with Age and Parkinson's Disease. Hints to Rehabilitation. Front Neurol. (2019) 10:532. 10.3389/fneur.2019.00532 31178816 PMC6543918

[4] Earhart GM. Dynamic control of posture across locomotor tasks. Mov Disord. (2013) 28:1501–8. 10.1002/mds.25592 24132838 PMC3801425

[5] Hase K Stein RB. Turning strategies during human walking. J Neurophysiol. (1999) 81:2914–22. 10.1152/jn.1999.81.6.2914 10368408

[6] Patla AE Adkin A Ballard T. Online steering: coordination and control of body center of mass, head and body reorientation. Exp Brain Res. (1999) 129:629–34. 10.1007/s002210050932 10638436

[7] Courtine G Schieppati M. Human walking along a curved path. I. Body trajectory, segment orientation and the effect of vision. Eur J Neurosci. (2003) 18:177–90. 10.1046/j.1460-9568.2003.02736.x 12859351

[8] Courtine G Schieppati M. Human walking along a curved path. II. Gait features and EMG patterns. Eur J Neurosci. (2003) 18:191–205. 10.1046/j.1460-9568.2003.02737.x 12859352

[9] Schmid M De Nunzio AM Schieppati M. Trunk muscle proprioceptive input assists steering of locomotion. Neurosci Lett. (2005) 384:127–32. 10.1016/j.neulet.2005.04.059 15885899

[10] Courtine G Schieppati M. Tuning of a basic coordination pattern constructs straight-ahead and curved walking in humans. J Neurophysiol. (2004) 91:1524–35. 10.1152/jn.00817.2003 14668296

[11] Ivanenko Y Gurfinkel VS. Human postural control. Front Neurosci. (2018) 12:171. 10.3389/fnins.2018.00171 29615859 PMC5869197

[12] Chia Bejarano N Pedrocchi A Nardone A Schieppati M Baccinelli W Monticone M. Tuning of muscle synergies during walking along rectilinear and curvilinear trajectories in humans. Ann Biomed Eng. (2017) 45:1204–18. 10.1007/s10439-017-1802-z 28144794

[13] Lewallen LK Srivastava S Kautz SA Neptune RR. Assessment of turning performance and muscle coordination in individuals post-stroke. J Biomech. (2021) 114:110113. 10.1016/j.jbiomech.2020.110113 33338757 PMC7874524

[14] Turcato AM Godi M Giordano A Schieppati M Nardone A. The generation of centripetal force when walking in a circle: insight from the distribution of ground reaction forces recorded by plantar insoles. J Neuroeng Rehabil. (2015) 12:4. 10.1186/1743-0003-12-4 25576354 PMC4325939

[15] Schieppati M. Coordination entre la posture et le movement. Le cas de la marche en courbe. In: Paillard T, editor. Posture et Equilibration Humaines. Paris: De Boeck Superieur (2016). p. 131–40.

[16] Paquette MR Fuller JR Adkin AL Vallis LA. Age-related modifications in steering behavior: effects of base-of-support constraints at the turn point. Exp Brain Res. (2008) 190:1–9. 10.1007/s00221-008-1448-z 18553073

[17] Mancini M Schlueter H El-Gohary M Mattek N Duncan C Kaye J. Continuous monitoring of turning mobility and its association to falls and cognitive function: a pilot study. J Gerontol A Biol Sci Med Sci. (2016) 71:1102–8. 10.1093/gerona/glw019 26916339 PMC5007616

[18] Thigpen MT Light KE Creel GL Flynn SM. Turning difficulty characteristics of adults aged 65 years or older. Phys Ther. (2016) 80:1174–87. 10.1093/ptj/80.12.1174 11087304

[19] Gordt K Müller C Gerhardy T Schwenk M. Influence of dual-tasking on straight ahead and curved walking in older adults. Z Gerontol Geriatr. (2019) 52:673–9. 10.1007/s00391-018-01482-3 30467671

[20] Gulley E Ayers E Verghese J. A comparison of turn and straight walking phases as predictors of incident falls. Gait Posture. (2020) 79:239–43. 10.1016/j.gaitpost.2020.05.002 32450510 PMC7299744

[21] Podsiadlo D Richardson S. The timed “Up & Go”: a test of basic functional mobility for frail elderly persons. J Am Geriatr Soc. (1991) 39:142–8. 10.1111/j.1532-5415.1991.tb01616.x 1991946

[22] Dotov DG Bardy BG Dalla Bella S. The role of environmental constraints in walking: effects of steering and sharp turns on gait dynamics. Sci Rep. (2016) 6:28374. 10.1038/srep28374 27345577 PMC4937443

[23] Guglielmetti S Nardone A De Nunzio AM Godi M Schieppati M. Walking along circular trajectories in Parkinson's disease. Mov Disord. (2009) 24:598–604. 10.1002/mds.22424 19117359

[24] Spildooren J Vercruysse S Desloovere K Vandenberghe W Kerckhofs E Nieuwboer A. Freezing of gait in Parkinson's disease: the impact of dual-tasking and turning. Mov Disord. (2010) 25:2563–70. 10.1002/mds.23327 20632376

[25] Hunter SW Divine A. The effect of walking path configuration on gait in adults with Alzheimer's dementia. Gait Post. (2018) 64:226–9. 10.1016/j.gaitpost.2018.06.118 29940482

[26] Herssens N Verbecque E Hallemans A Vereeck L Van Rompaey V Saeys W. Do spatiotemporal parameters and gait variability differ across the lifespan of healthy adults? A systematic review. Gait Post. (2013) 64:181–90. 10.1016/j.gaitpost.2018.06.012 29929161

[27] Benedetti MG Beghi E De Tanti A Cappozzo A Basaglia N Cutti AG. SIAMOC position paper on gait analysis in clinical practice: general requirements, methods and appropriateness. Results of an Italian consensus conference. Gait Post. (2017) 58:252–60. 10.1016/j.gaitpost.2017.08.003 28825997

[28] Belluscio V Bergamini E Tramontano M Formisano R Buzzi MG Vannozzi G. Does curved walking sharpen the assessment of gait disorders? An instrumented approach based on wearable inertial sensors. Sensors. (2020) 20:5244. 10.3390/s20185244 32937877 PMC7570481

[29] Leach JM Mellone S Palumbo P Bandinelli S Chiari L. Natural turn measures predict recurrent falls in community-dwelling older adults: a longitudinal cohort study. Sci Rep. (2013) 8:4316. 10.1038/s41598-018-22492-6 29531284 PMC5847590

[30] Tegner Y Lysholm J Lysholm M Gillquist J. A performance test to monitor rehabilitation and evaluate anterior cruciate ligament injuries. Am J Sports Med. (1986) 14:156–9. 10.1177/036354658601400212 3717488

[31] Risberg MA Ekeland A. Assessment of functional tests after anterior cruciate ligament surgery. J Orthop Sports Phys Ther. (1994) 19:212–7. 10.2519/jospt.1994.19.4.212 8173569

[32] Shkuratova N Morris ME Huxham F. Effects of age on balance control during walking. Arch Phys Med Rehabil. (2004) 85:582–8. 10.1016/j.apmr.2003.06.021 15083433

[33] Hess RJ Brach JS Piva SR VanSwearingen. Walking skill can be assessed in older adults: validity of the Figure-of-8 Walk Test. Phys Ther. (2010) 90:89–99. 10.2522/ptj.20080121 19959654 PMC2802825

[34] Bland K Lowry K Krajek A Woods T VanSwearingen J. Spatiotemporal variability underlying skill in curved-path walking. Gait Post. (2019) 67:137–41. 10.1016/j.gaitpost.2018.10.001 30336347

[35] Schack J Mirtaheri P Steen H Gjøvaag T. Assessing mobility for persons with lower limb amputation: the Figure-of-Eight Walk Test with the inclusion of two novel conditions. Disabil Rehabil. (2019) 17:1–10. 10.1080/09638288.2019.1662495 31526078

[36] O'Keefe DJ. Brief report: post hoc power, observed power, a priori power, retrospective power, prospective power, achieved power: sorting out appropriate uses of statistical power analyses. Commun Methods Meas. (2007) 1:291–9. 10.1080/19312450701641375

[37] Georgiev GZ. Power & Sample Size Calculator. (2021). Available online at: https://www.gigacalculator.com/calculators/power-sample-size-calculator.php (accessed January 11, 2021).

[38] Forte R De Vito G Boreham CAG. Reliability of walking speed in basic and complex conditions in healthy, older community-dwelling individuals. Aging Clin Exp Res. (2021) 33:311–7. 10.1007/s40520-020-01543-x 32277431

[39] Rosso AL Metti AL Faulkner K Brach JS Studenski SA Redfern M. Associations of usual pace and complex task gait speeds with incident mobility disability. J Am Geriatr Soc. (2019) 67:2072–6. 10.1111/jgs.16049 31318048 PMC6800783

[40] Turcato AM Godi M Giardini M Arcolin I Nardone A Giordano A. Abnormal gait pattern emerges during curved trajectories in high-functioning Parkinsonian patients walking in line at normal speed. PLoS ONE. (2018) 13:e0197264. 10.1371/journal.pone.0197264 29750815 PMC5947908

[41] Nolasco LA Silverman AK Gates DH. Whole-body and segment angular momentum during 90-degree turns. Gait Post. (2019) 70:12–19. 10.1016/j.gaitpost.2019.02.003 30776765

[42] Leirós-Rodríguez R García-Liñeira J Soto-Rodríguez A García-Soidán JL. Percentiles and reference values for accelerometric gait assessment in women aged 50-80 years. Brain Sci. (2020) 10:832. 10.3390/brainsci10110832 PMC7695338 33182373

[43] Bohannon RW Wang YC. Four-meter gait speed: normative values and reliability determined for adults participating in the NIH toolbox study. Arch Phys Med Rehabil. (2019) 100:509–13. 10.1016/j.apmr.2018.06.031 30092204 PMC6363908

[44] Virmani T Gupta H Shah J Larson-Prior L. Objective measures of gait and balance in healthy non-falling adults as a function of age. Gait Post. (2018) 65:100–5. 10.1016/j.gaitpost.2018.07.167 30558914 PMC9115806

[45] Kawai H Taniguchi Y Seino S Sakurai R Osuka Y Obuchi S. Reference values of gait parameters measured with a plantar pressure platform in community-dwelling older Japanese adults. Clin Interv Aging. (2019) 14:1265–76. 10.2147/CIA.S213216 31371932 PMC6636431

[46] Lau LK Wee SL Pang WJB Chen KK Abdul Jabbar K Yap PLK. Reference values of gait speed and gait spatiotemporal parameters for a South East Asian Population: The Yishun Study. Clin Interv Aging. (2020) 15:1753–65. 10.2147/CIA.S270407 33061327 PMC7522423

[47] Peters DM Fritz SL Krotish DE. Assessing the reliability and validity of a shorter walk test compared with the 10-Meter Walk Test for measurements of gait speed in healthy, older adults. J Geriatr Phys Ther. (2013) 36:24–30. 10.1519/JPT.0b013e318248e20d 22415358

[48] Krumpoch S Lindemann U Rappl A Becker C Sieber CC Freiberger E. The effect of different test protocols and walking distances on gait speed in older persons. Aging Clin Exp Res. (2021) 33:141–6. 10.1007/s40520-020-01703-z 32930990 PMC7897617

[49] Winter DA Patla AE Frank JS Walt SE. Biomechanical walking pattern changes in the fit and healthy elderly. Phys Ther. (1990) 70:340–7. 10.1093/ptj/70.6.340 2345777

[50] Morris ME Iansek R Matyas TA Summers JJ. Ability to modulate walking cadence remains intact in Parkinson's disease. J Neurol Neurosurg Psychiatry. (1994) 57:1532–4. 10.1136/jnnp.57.12.1532 7798986 PMC1073238

[51] Lindemann U Schwickert L Becker C Gross M Nolte R Klenk J. Estimate of gait speed by using persons' walk ratio or step-frequency in older adults. Aging Clin Exp Res. (2021). 10.1007/s40520-021-01832-z. [Epub ahead of print]. 33778931

[52] Sekiya N Nagasaki H. Reproducibility of the walking patterns of normal young adults: test-retest reliability of the walk ratio (step-length/step-rate). Gait Post. (1998) 7:225–7. 10.1016/S0966-6362(98)00009-5 10200388

[53] Terrier P Reynard F. Effect of age on the variability and stability of gait: a cross-sectional treadmill study in healthy individuals between 20 and 69 years of age. Gait Post. (2015) 41:170–4. 10.1016/j.gaitpost.2014.09.024 25455699

[54] Zijlstra A de Bruin ED Bruins N Zijlstra W. The step length-frequency relationship in physically active community-dwelling older women. Eur J Appl Physiol. (2008) 104:427–34. 10.1007/s00421-008-0795-6 18553099

[55] Egerton T Danoudis M Huxham F Iansek R. Central gait control mechanisms and the stride length - cadence relationship. Gait Post. (2011) 34:178–82. 10.1016/j.gaitpost.2011.04.006 21550245

[56] Murakami R Otaka Y. Estimated lower speed boundary at which the walk ratio constancy is broken in healthy adults. J Phys Ther Sci. (2017) 29:722–5. 10.1589/jpts.29.722 28533617 PMC5430280

[57] Rota V Perucca L Simone A Tesio L. Walk ratio (step length/cadence) as a summary index of neuromotor control of gait: application to multiple sclerosis. Int J Rehabil Res. (2011) 34:265–9. 10.1097/MRR.0b013e328347be02 21629125

[58] Courtine G Papaxanthis C Schieppati M. Coordinated modulation of locomotor muscle synergies constructs straight-ahead and curvilinear walking in humans. Exp Brain Res. (2006) 170:320–35. 10.1007/s00221-005-0215-7 16328271

[59] Neptune RR McGowan CP. Muscle contributions to frontal plane angular momentum during walking. J Biomech. (2016) 49:2975–81. 10.1016/j.jbiomech.2016.07.016 27522538 PMC5056157

[60] Nakakubo S Doi T Makizako H Tsutsumimoto K Hotta R Kurita S. Association of walk ratio during normal gait speed and fall in community-dwelling elderly people. Gait Post. (2018) 66:151–4. 10.1016/j.gaitpost.2018.08.030 30195217

[61] Kalron A Menascu S Givon U Dolev M Achiron A. Is the walk ratio a window to the cerebellum in multiple sclerosis? A structural magnetic resonance imaging study. Eur J Neurol. (2020) 27:454–60. 10.1111/ene.14119 31696586

[62] Caballero-Mora MA Rodríguez Mañas L Valdés-Aragonés M García-Sánchez I Alonso-Bouzon C Castro Rodríguez M. Factors associated with impairment in gait speed in older people with clinically normal gait. A cross-sectional study. Aging Clin Exp Res. (2020) 32:1043–8. 10.1007/s40520-019-01187-6 30989508

[63] Fitzgerald C Thomson D Zebib A Clothier PJ Gupta A. A comparison of gait stability between younger and older adults while head turning. Exp Brain Res. (2020) 238:1871–83. 10.1007/s00221-020-05846-3 32529291

[64] Osoba MY Rao AK Agrawal SK Lalwani AK. Balance and gait in the elderly: a contemporary review. Laryngosc Investig Otolaryngol. (2019) 4:143–53. 10.1002/lio2.252 30828632 PMC6383322

[65] Uiga L Cheng KC Wilson MR Masters RS Capio CM. Acquiring visual information for locomotion by older adults: a systematic review. Ageing Res Rev. (2015) 20:24–34. 10.1016/j.arr.2014.12.005 25576650

[66] Welch SA Ward RE Kurlinski LA Kiely DK Goldstein R VanSwearingen J. Straight and curved path walking among older adults in primary care: associations with fall-related outcomes. PM R. (2016) 8:754–60. 10.1016/j.pmrj.2015.12.004 26733078 PMC4925327

[67] Egerton T Brauer SG Cresswell AG. Fatigue after physical activity in healthy and balance-impaired elderly. J Aging Phys Act. (2009) 17:89–105. 10.1123/japa.17.1.89 19299841

[68] Zhou Y Romijnders R Hansen C Campen JV Maetzler W Hortobágyi T. The detection of age groups by dynamic gait outcomes using machine learning approaches. Sci Rep. (2020) 10:4426. 10.1038/s41598-020-61423-2 32157168 PMC7064519

[69] Zhang Y Smeets JBJ Brenner E Verschueren S Duysens J. Effects of ageing on responses to stepping-target displacements during walking. Eur J Appl Physiol. (2021) 121:127–40. 10.1007/s00421-020-04504-4 32995959 PMC7815571

[70] Neumann DA. Kinesiology of the hip: a focus on muscular actions. J Orthop Sports Phys Ther. (2010) 40:82–94. 10.2519/jospt.2010.3025 20118525

[71] Flack NA Nicholson HD Woodley SJ. Thje anatomy of the hip abductor muscles. Clin Anat. (2014) 27:241–53. 10.1002/ca.22248 23625344

[72] Sozzi S Nardone A Crisafulli O Schieppati M. Podokinetic after-rotation is transiently enhanced or reversed by unilateral axial muscle proprioceptive stimulation. Neural Plast. (2019) 2019:7129279. 10.1155/2019/7129279 30984256 PMC6432728

[73] Krause DA Neuger MD Lambert KA Johnson AE DeVinny HA Hollman JH. Effects of examiner strength on reliability of hip-strength testing using a handheld dynamometer. J Sport Rehabil. (2014) 23:56–64. 10.1123/JSR.2012-0070 24231811

[74] Bayot M Dujardin K Dissaux L Tard C Defebvre L Bonnet CT. Can dual-task paradigms predict falls better than single task? - A systematic literature review. Neurophysiol Clin. (2020) 50:401–40. 10.1016/j.neucli.2020.10.008 33176988

[75] Monaco V Ghionzoli A Micera S. Age-related modifications of muscle synergies and spinal cord activity during locomotion. J Neurophysiol. (2010) 104:2092–102. 10.1152/jn.00525.2009 20685924

[76] Baloh RW Ying SH Jacobson KM. A longitudinal study of gait and balance dysfunction in normal older people. Arch Neurol. (2003) 60:835–9. 10.1001/archneur.60.6.835 12810488

[77] Chen IH Yang YR Chan RC Wang RY. Turning-based treadmill training improves turning performance and gait symmetry after stroke. Neurorehabil Neural Repair. (2014) 28:45–55. 10.1177/1545968313497102 23897905

[78] Godi M Giardini M Nardone A Turcato AM Caligari M Pisano F. Curved walking rehabilitation with a rotating treadmill in patients with Parkinson's Disease: a proof of concept. Front Neurol. (2017) 8:53. 10.3389/fneur.2017.00053 28293213 PMC5329030

[79] Paquette C Franzén E Jones GM Horak FB. Walking in circles: navigation deficits from Parkinson's disease but not from cerebellar ataxia. Neuroscience. (2011) 190:177–83. 10.1016/j.neuroscience.2011.06.020 21704129 PMC3156363

[80] Allali G Launay CP Blumen HM Callisaya ML De Cock AM Kressig RW. Biomathics consortium falls, cognitive impairment, and gait performance: results from the GOOD initiative. J Am Med Dir Assoc. (2017) 18:335–40. 10.1016/j.jamda.2016.10.008 27914848 PMC5366266

[81] Mirelman A Bonato P Camicioli R Ellis TD Giladi N Hamilton JL. Gait impairments in Parkinson's disease. Lancet Neurol. (2019) 18:697–708. 10.1016/S1474-4422(19)30044-4 30975519

[82] Wilson J Allcock L Mc Ardle R Taylor JP Rochester L. The neural correlates of discrete gait characteristics in ageing: a structured review. Neurosci Biobehav Rev. (2019) 100:344–69. 10.1016/j.neubiorev.2018.12.017 30552912 PMC6565843

